# P-1890. Building the HIV Workforce through Integrating the National HIV Curriculum into Health Professions Programs in the United States

**DOI:** 10.1093/ofid/ofaf695.2059

**Published:** 2026-01-11

**Authors:** Cornelia Wagner, Ricardo Rivero, Jonathan Stacks, Natacha Pierre, Blake Max

**Affiliations:** University of Illinois Chicago, Wilmette, IL; University of Illinois Chicago, Wilmette, IL; University of Illinois Chicago, Wilmette, IL; University of Illinois Chicago, Wilmette, IL; University of Illinois Chicago Retzky College of Pharmacy, Chicago, Illinois

## Abstract

**Background:**

To address national shortages in the HIV clinical workforce, the Midwest AIDS Training and Education Center (MATEC) at the University of Illinois Chicago integrated the National HIV Curriculum (NHC) into the existing curricula of 24 health professions programs (HPPs) to build a pipeline of future healthcare professionals who are adequately trained in HIV care and prevention.

**Figure:**
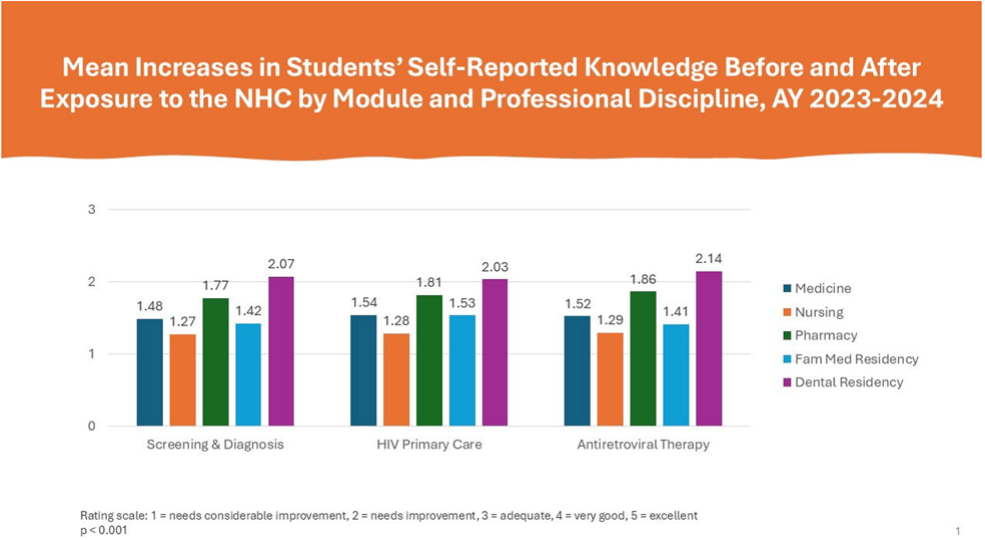

**Figure:**
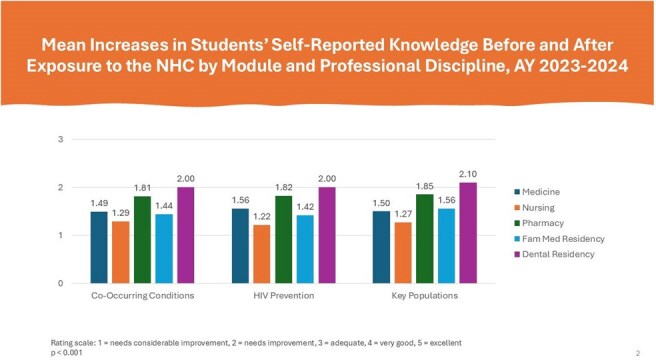

**Methods:**

During a national recruitment effort, 24 HPPs in medicine (4), nursing (7), pharmacy (4), family medicine residency (6), and dental residency (3) were carefully selected for participation in the project. Participating faculty completed pre- and post-integration surveys for each course in which they integrated content from the NHC to document the integration process. Students and residents completed retrospective pre-post assessments using five-point Likert scales to measure changes in knowledge of HIV prevention and care and intent to provide care to people with HIV and at high risk for HIV. Student data were analyzed using Wilcoxon signed-rank tests.

**Figure:**
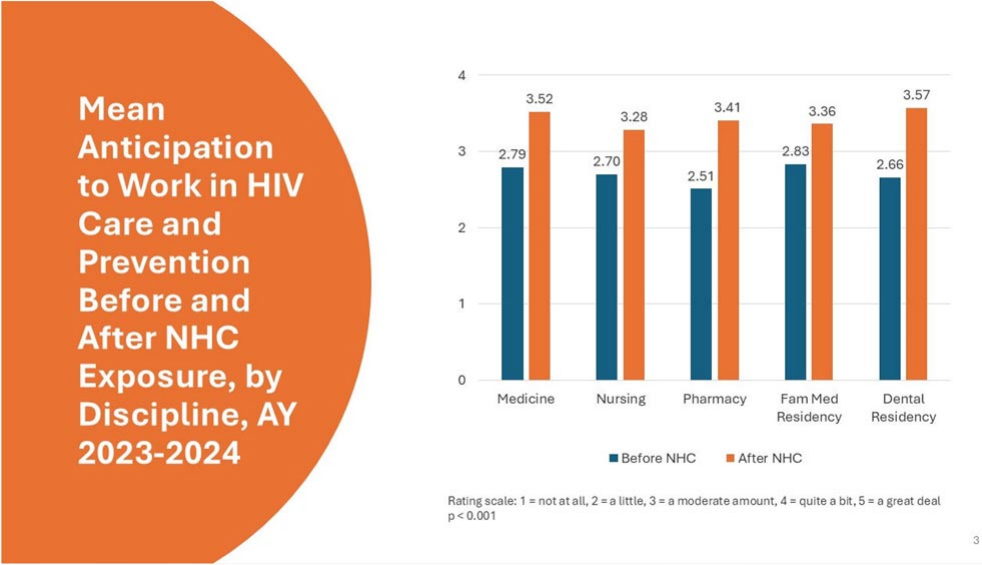

**Figure:**
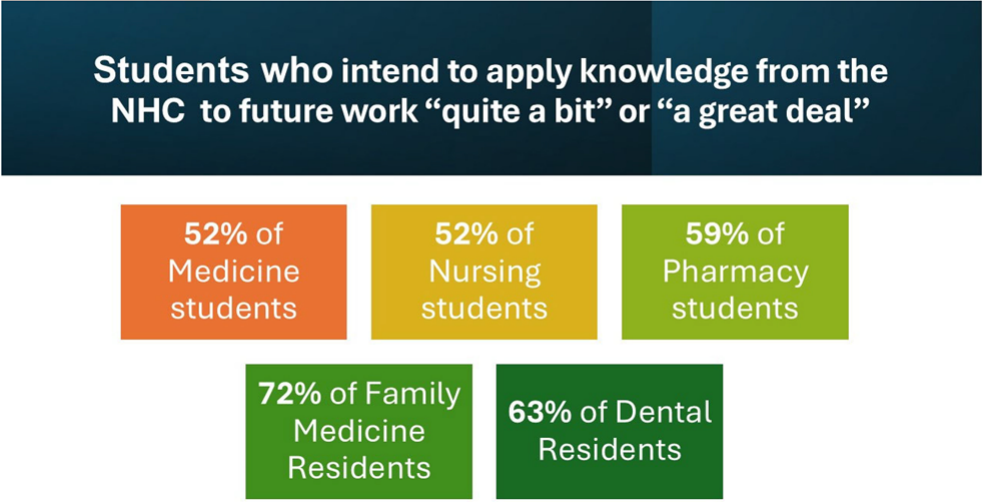

**Results:**

During the academic year 2023-2024, faculty from participating HPPs integrated the NHC into 95 courses with a combined enrollment of 4,236 students and residents. On average, learners across all five health disciplines reported statistically significant increases (*p*< 0.001) in knowledge of HIV prevention and care and in intent to work with people with HIV in their future careers. Seventy-two (72) percent of family medicine, 63% of dental, 59% of pharmacy, and 52% of nursing and medicine respondents indicated intent to apply knowledge acquired from the NHC ‘quite a bit’ or ‘a great deal’ in their future careers.

**Conclusion:**

The integration of the NHC into the existing curricula of HPPs can have a positive impact on expanding the HIV workforce.

**Disclosures:**

All Authors: No reported disclosures

